# Prognostic nutritional index at discharge as a practical bedside tool for long-term all-cause mortality risk in pneumonia-induced sepsis survivors

**DOI:** 10.3389/fnut.2026.1813273

**Published:** 2026-05-28

**Authors:** Zhenwei Zhai, Junyu He, Jingxia Sun, Wenxin Chu, Rongyan Wei, Yanni Tan, Haolun Wang, Jie Lu, Zhengming Li, Jianhao Huang, Quan Lu, Wensheng Lu

**Affiliations:** 1 Department of Endocrinology and Metabolism, National Key Clinical Construction Specialty, Guangxi Academy of Medical Sciences & The People's Hospital of Guangxi Zhuang Autonomous Region, Nanning, Guangxi, China; 2 Department of Endocrinology and Respiratory, The Third People's Hospital of Nanning, Nanning, Guangxi, China; 3 Clinical Physician Training Base, Guangxi Academy of Medical Sciences & The People's Hospital of Guangxi Zhuang Autonomous Region, Nanning, Guangxi, China

**Keywords:** long-term all-cause mortality, nutrition-immunoinflammation markers, pneumonia-induced sepsis, prognostic nutritional index (PNI), sepsis survivor

## Abstract

**Objectives:**

Existing research has predominantly focused on short-term outcomes of sepsis during intensive care unit (ICU) admission, with limited evidence on long-term all-cause mortality. This multicenter ambispective longitudinal cohort study aimed to evaluate the prognostic value of the prognostic nutritional index (PNI), a composite marker reflecting nutritional and immune-inflammatory status, for long-term all-cause mortality in pneumonia-induced sepsis survivors.

**Methods:**

A total of 461 pneumonia-induced sepsis survivors with recurrence-free status for at least 3 months post-discharge were retrospectively enrolled and prospectively followed for a median of 36 months. Participants were stratified into tertiles based on discharge PNI scores. The association between PNI and long-term all-cause mortality was assessed, and its prognostic performance was compared with established indicators, including the Controlling Nutritional Status (CONUT) score, modified Glasgow Prognostic Score (mGPS), blood urea nitrogen-to-albumin ratio (BAR), neutrophil-to-lymphocyte ratio (NLR), and platelet-to-lymphocyte ratio (PLR).

**Results:**

Restricted cubic spline (RCS) analysis revealed an inverted L-shaped, dose-dependent negative association between PNI and long-term all-cause mortality risk (*p* for overall < 0.001). Multivariate Cox regression analysis showed that participants in the lowest PNI tertile had a 54.8% higher mortality risk than those in the highest tertile (*p* for trend = 0.043). Subgroup analyses confirmed consistent prognostic value across diverse populations (all interaction *p*-values > 0.05). Kaplan-Meier analysis demonstrated that lower PNI levels were significantly associated with higher cumulative mortality (log-rank, *p* < 0.001). Multivariate linear regression further indicated that each one-tertile increase in PNI corresponded to a quantifiable reduction [calculated as |β| × tertile interval (5.9)] in related nutritional and inflammatory markers, in model III, including a reduction of 1.0585 in COUNT score (β = −0.1794), 0.1398 in mGPS (β = −0.0237), 0.0378 in BAR (β = −0.0064), 45.6619 in PLR (β = −7.7393), and 2.8951 in NLR (β = −0.4907) (all *p* < 0.001). Receiver operating characteristic (ROC) curve analysis identified a PNI clinical diagnostic cutoff of 39.00 for long-term all-cause mortality, with an area under the curve (AUC) of 0.672 (*p* < 0.001) and a sensitivity of 0.64, outperforming the established indicators.

**Conclusions:**

These findings suggest that PNI may serve as a practical bedside tool for estimating long-term post-hospital all-cause mortality among survivors of pneumonia-induced sepsis, supporting personalized clinical decision-making and routine care as an auxiliary prognostic indicator.

## Highlights

- Why did we conduct this study? Sepsis survivors remain at elevated risk of long-term all-cause mortality after hospital discharge.
- What specific question were we seeking to address? Can the prognostic nutritional index (PNI), as a composite marker reflecting nutritional, immune, and inflammatory status, predict long-term all-cause mortality in survivors of pneumonia-induced sepsis?
- What are the findings? Patients in the lowest PNI tertile exhibited a 54.8% higher risk of mortality compared to those in the highest tertile. An inverted L-shaped, dose-dependent negative association was observed between PNI levels and long-term all-cause mortality risk.
- What is the significance of these results? PNI may serve as a simple, cost-effective bedside tool for predicting long-term all-cause mortality in survivors of pneumonia-induced sepsis. It could support personalized clinical decision-making and routine care by functioning as an auxiliary prognostic indicator.

## 1 Introduction

Sepsis remains the leading cause of death in intensive care units (ICUs) globally (1). Although advances in treatment strategies have significantly reduced in-hospital mortality in recent years, survivors often face considerable long-term burdens (2). Research shows that long-term all-cause mortality risk in sepsis survivors remains markedly higher than in the general population (3), along with frequent rehospitalizations (4), lasting functional limitations, and diminished quality of life (5). Together, these consequences pose a major public health challenge worldwide (6).

Established clinical scoring systems such as the Acute Physiology and Chronic Health Evaluation (APACHE) II score (https://www.mdcalc.com/calc/1868/apache-ii-score), the Sequential Organ Failure Assessment (SOFA) score (used in ICUs) (https://www.mdcalc.com/calc/691/sequential-organ-failure-assessment-sofa-score), and the quick SOFA (qSOFA) score (applied in non-ICU hospital settings) (https://www.mdcalc.com/calc/2654/qsofa-quick-sofa-score-sepsis) are typically calculated from the worst test values recorded within the first 24 h of ICU admission or hospitalization. Designed initially as mortality prediction tools for clinicians to assess patient conditions or communicate with families, they were not intended to guide clinical decisions during ICUs or hospital care.

Malnutrition and immune-inflammatory dysfunction persist after sepsis, forming a vicious cycle within the “persistent inflammation-immunosuppression-catabolism syndrome (PICS)” that mutually exacerbates each other (7). Hypoalbuminemia not only reflects poor nutritional reserve but is also associated with inflammatory activity (8), capillary leakage (9), and immunosuppression (10). Lymphocytopenia is a key hallmark of sepsis-induced immunosuppression and is strongly associated with increased risks of secondary infections and death (11–13). Although several markers positively related to mortality, including the Controlling Nutritional Status (CONUT) score, blood urea nitrogen-to-albumin ratio (BAR), platelet-to-lymphocyte ratio (PLR), and neutrophil-to-lymphocyte ratio (NLR), have been used to predict short-term sepsis mortality risk, each has limitations: the CONUT score reflects nutritional status but does not fully account for immune-inflammatory status (14); the BAR incorporates renal function and albumin but lacks immune and inflammatory information (15); the PLR (16) and NLR (17) indicate inflammatory status but omit nutritional assessment. The modified Glasgow Prognostic Score (mGPS), based on serum C-reactive protein (CRP) and albumin levels, suggests that higher scores reflect more severe systemic inflammation and poorer nutritional reserve; however, prior research on mGPS has primarily involved cancer patients (18). Thus, studies focusing on long-term all-cause mortality risk in sepsis survivors after hospital discharge remain limited.

The Prognostic Nutritional Index (PNI), derived from serum albumin and lymphocyte count (19), integrates both a nutrition- and inflammation-related marker (albumin) and an immune status indicator (lymphocytes) (20). Initially developed for patients undergoing gastrointestinal cancer surgery, PNI has since demonstrated prognostic value in various cancers (21–23), cardiovascular diseases (24), and surgical patients (25). Preliminary studies have also explored its role in the acute phase of sepsis (26). However, its efficacy in predicting long-term adverse outcomes based on ICU discharge status remains thoroughly unexplored. This study aims to evaluate whether PNI measured at ICU discharge can effectively integrate nutritional and immune parameters to predict long-term all-cause mortality in sepsis survivors. Using a multicenter, ambispective longitudinal cohort design, we will assess the predictive performance of PNI for long-term all-cause mortality risk in survivors of pneumonia-induced sepsis and compare it with established indicators (CONUT, mGPS, BAR, NLR, and PLR). The goal is to provide clinicians with a practical, cost-effective bedside tool for prognostic evaluation in this vulnerable patient population.

## 2 Materials and methods

### 2.1 Ethics approval

The study protocol received approval from the Ethics Committee of the Guangxi Academy of Medical Sciences & the People's Hospital of Guangxi Zhuang Autonomous Region (Approval No. Ethics-KY-IIT-2023-60) and the Ethics Committee of the Third People's Hospital of Nanning (Approval No. 2025081). Before enrollment, written informed consent was obtained from all participants. The research was conducted in compliance with the ethical principles outlined in the Declaration of Helsinki.

### 2.2 Study design and participant selection

**Figure 1:**
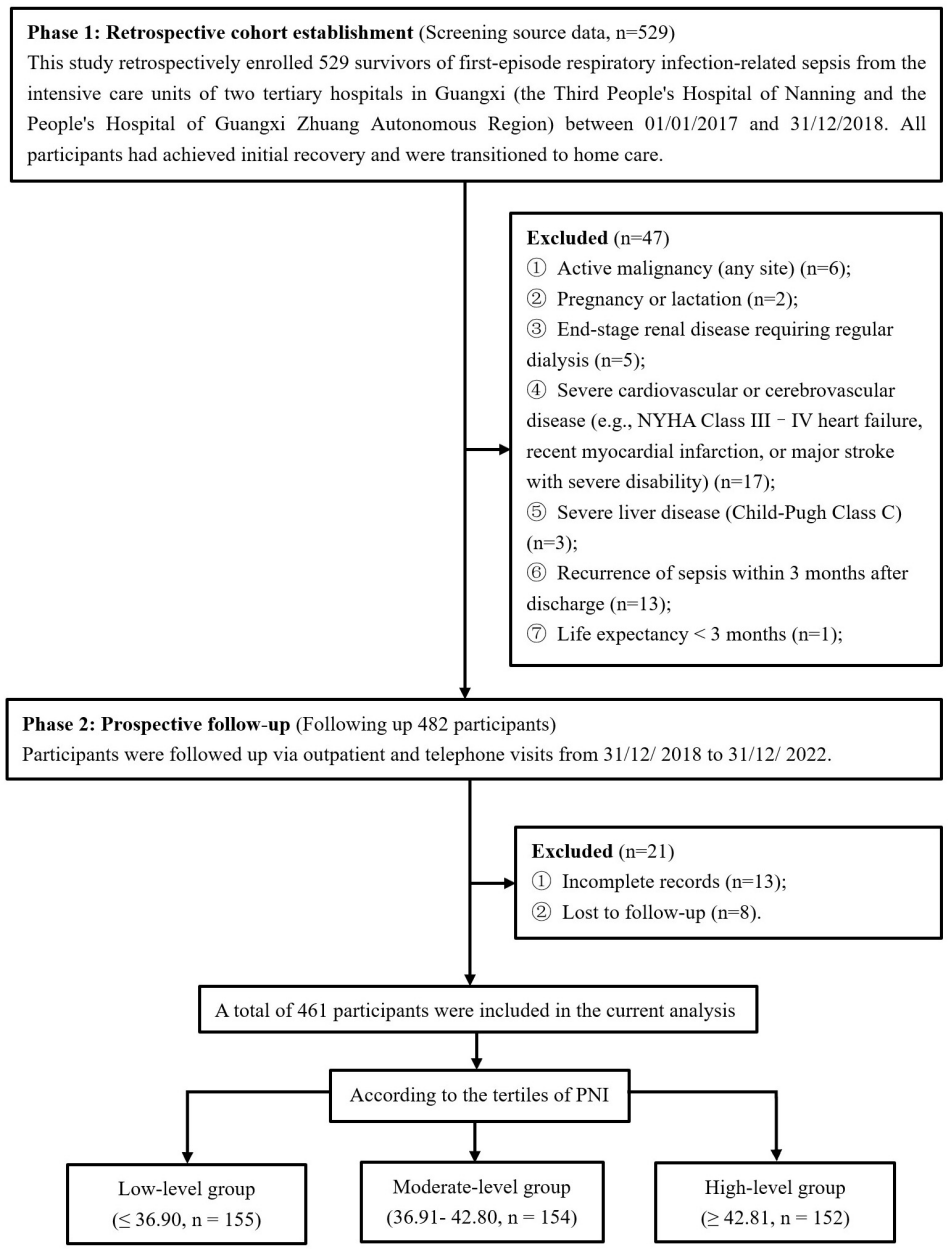
Flowchart of the study design and participant screening strategy. The multicenter ambispective longitudinal cohort design comprised two phases: Phase 1 (retrospective cohort) and Phase 2 (prospective follow-up). A total of 461 participants were included in the analysis. Participants were divided into low (≤ 36.90, *n* = 155), moderate (36.90–42.81, *n* = 154), and high (≥ 42.81, *n* = 152) PNI tertiles.

The study design and participant screening strategy are illustrated in Figure 1. This study employed a multicenter, ambispective longitudinal cohort design, combining retrospective data collection from electronic medical records with a prospective follow-up phase to assess long-term all-cause mortality. A retrospective cohort was first established, including 529 survivors of pneumonia-induced sepsis who were discharged from the ICUs of the Third People's Hospital of Nanning and the People's Hospital of Guangxi Zhuang Autonomous Region, between January 1, 2017, and December 31, 2018. All enrolled participants were sepsis recurrence-free for at least 3 months after discharge. After applying the exclusion criteria, 47 cases were removed, leaving 482 patients who entered the prospective phase. Prospective follow-up for all-cause mortality endpoints was conducted from December 31, 2018, to December 31, 2022, with a median of 36 months, via outpatient visits and telephone interviews. Using a complete-case analysis, an additional 21 patients were excluded for incomplete data or loss to follow-up, yielding a final cohort of 461 patients. Inclusion criteria were: (1) age ≥ 18 years; (2) sepsis diagnosis according to the Sepsis-3 criteria (27); (3) discharge from the ICU to home care in a clinically stable condition; and (4) availability of the most recent complete laboratory data before discharge. Exclusion criteria included: (1) active malignancy (any site; *n* = 6); (2) pregnancy or lactation (*n* = 2); (3) end-stage renal disease requiring regular dialysis (*n* = 5); (4) severe cardiovascular or cerebrovascular disease, such as New York Heart Association (NYHA) Class III–IV heart failure, recent myocardial infarction, or major stroke causing severe disability (*n* = 17); (5) severe liver disease (Child-Pugh Class C; *n* = 3); (6) recurrence of sepsis within 3 months after discharge (*n* = 13); (7) life expectancy < 3 months (*n* = 1); and (8) incomplete clinical data (*n* = 13) or loss to follow-up (*n* = 8).

### 2.3 Data collection

This study used electronic medical records from two centers, the Third People's Hospital of Nanning and the People's Hospital of Guangxi Zhuang Autonomous Region, to obtain baseline demographic and serum biochemistry data from sepsis survivors who had reached clinical stability and were preparing for discharge to home care. To minimize variability, blood specimens were collected after an 8–12-h overnight fast on the day before discharge and delivered to the central laboratory within 1 h for automated analysis. Hematological parameters were measured using the Horiba ABX Pentra 120R system, and biochemical markers were measured using the Roche Cobas P800 platform. All-cause mortality data were obtained through retrospective record review and active follow-up. In line with a complete-case analytical approach, only subjects with nearly complete datasets (missingness < 5%) were retained; cases with missing data or lost to follow-up were excluded. A uniform, de-identified database was subsequently constructed to support further statistical evaluation.

### 2.4 Related definitions

Definitions in this study were established as follows: (1) Sepsis survivors were defined as patients meeting clinical discharge criteria who transitioned to home-based rehabilitation. Initial sepsis recovery was characterized by the absence of recurrence within 3 months post-discharge. (2) The endpoint was all-cause mortality. (3) Survival time was calculated from ICU admission until sepsis recurrence or the end of the study, whichever occurred earlier. (4) Patients alive at follow-up completion or experiencing recurrence more than 3 months after enrollment were treated as censored. (5) Baseline was defined as the discharge date following initial sepsis resolution. (6) The CONUT score was derived using the method outlined in Supplementary material Figure 1 (14). (7) BAR = blood urea nitrogen concentration (mg/dl)/serum albumin concentration (g/dl) (15). (8) PLR= platelet count (× 10^9^/L)/absolute lymphocyte count (× 10^9^/L) (16). (9) NLR = absolute neutrophil count (× 10^9^/L)/absolute lymphocyte count (× 10^9^/L) (17). (10) mGPS was derived from serum C-reactive protein (CRP) and albumin measurements. Patients with CRP ≤ 10 mg/L received a score of 0. Those with CRP > 10 mg/L were assigned 1 point if albumin was ≥35 g/L, and 2 points if albumin was < 35 g/L. Consequently, a higher mGPS indicates more pronounced systemic inflammation and worse nutritional reserve status (18). (11) PNI = serum albumin (g/L) + [5 × absolute lymphocyte count (× 10^9^/L)] (19).

### 2.5 Statistical analysis

All statistical analyses were performed with SPSS 26.0 (IBM Corp., Armonk, NY, USA) and R 4.2.2 (R Foundation, Vienna, Austria). Continuous non-normally distributed variables (not following a Gaussian distribution) were expressed as medians and interquartile ranges (IQRs), while categorical variables were reported as frequencies and percentages. Group comparisons used independent-samples *t*-tests for normally distributed continuous variables, Kruskal–Wallis *H*-tests (with Dunn's *post hoc* tests and a Bonferroni correction) for non-normally distributed continuous variables, and chi-square tests for categorical variables. Risk factors were evaluated via multivariable Cox proportional hazards regression, and survival outcomes were compared using Kaplan–Meier curves with the log-rank test. Restricted cubic spline (RCS) models were applied to assess nonlinear relationships. The association between PNI and nutritional-immunoinflammatory status was estimated using multivariate linear regression. Predictive performance was evaluated with receiver operating characteristic (ROC) curve analysis. Internal validation included 5-fold cross-validation, a bootstrap-corrected procedure (1,000 repetitions), and calibration plot analysis. Baseline characteristics were generated using the R package tableone, and all figures were produced in R. Statistical significance was defined as a *P*-value < 0.05.

## 3 Results

### 3.1 Baseline characteristics of participants

The baseline characteristics of the study participants are summarized in Table 1. Of the initial cohort screened according to the study criteria, 461 sepsis survivors were included in the analysis. The overall median age was 68.0 years (IQR: 56.5–77.0). Participants were stratified into three groups by PNI tertiles: low (PNI ≤ 36.90, *n* = 155), moderate (PNI 36.90–42.81, *n* = 154), and high (PNI ≥ 42.81, *n* = 152). Significant intergroup differences (all *P* < 0.05) were observed in age, diabetes prevalence, all-cause mortality, and multiple laboratory measures including neutrophil (Neu), lymphocyte (Lym), red blood cell (RBC), platelet, hemoglobin (Hb), C-reactive protein (CRP), prothrombin time (PT), thrombin time (TT), fibrinogen (Fib), total bilirubin (TB), direct bilirubin (DBIL), aspartate aminotransferase (AST), total protein (TP), albumin (ALB), creatinine (Cr), total cholesterol (TC), triglycerides (TG), high-density lipoprotein cholesterol (HDL-C), low-density lipoprotein cholesterol (LDL-C), PLR, NLR, COUNT score, BAR, mGPS. In contrast, no significant differences were found in gender, hypertension, white blood cell count (WBC), activated partial thromboplastin time (APTT), indirect bilirubin (IBIL), alanine aminotransferase (ALT), globulin (GLO), or uric acid (UA) (all *p* > 0.05).

**Table 1:** Baseline characteristics of participants.

Parameters	Overall	Low-PNI (≤ 36.90)	Moderate-PNI (36.90- 42.81)	High-PNI (≥42.81)	*p*-value
Demographic data					
N	461	155	154	152	< 0.001^*^
15.6-7.4,-1.3498pt Age, years	68.00 (58.00, 76.00)	72.00 (63.50, 79.00)	69.00 (60.00, 76.00)	64.00 (51.00, 71.00)	
**Gender**, ***n*** **(%)**					
Male	241 (52.28%)	78 (50.32%)	86 (55.84%)	77 (50.66%)	0.554
15.6-7.4,-1.3498pt Female	220 (47.72%)	77 (49.68%)	68 (44.16%)	75 (49.34%)	
**Hypertension**, ***n*** **(%)**					
No	367 (79.61%)	132 (85.16%)	119 (77.27%)	116 (76.32%)	0.107
15.6-7.4,-1.3498pt Yes	94 (20.39%)	23 (14.84%)	35 (22.73%)	36 (23.68%)	
**Diabetes**, ***n*** **(%)**					
No	305 (66.16%)	86 (55.48%)	109 (70.78%)	110 (72.37%)	0.003^*^
15.6-7.4,-1.3498pt Yes	156 (33.84%)	69 (44.52%)	45 (29.22%)	42 (27.63%)	
**CVD**, ***n*** **(%)**					
No	283 (61.39%)	85 (54.84%)	94 (61.04%)	104 (68.42%)	0.050
15.6-7.4,-1.3498pt Yes	178 (38.61%)	70 (45.16%)	60 (38.96%)	48 (31.58%)	
**All-cause mortality**, ***n*** **(%)**					
No	284 (61.61%)	66 (42.58%)	99 (64.29%)	119 (78.29%)	< 0.001^*^
15.6-7.4,-1.3498pt Yes	177 (38.39%)	89 (57.42%)	55 (35.71%)	33 (21.71%)	
**Laboratory test data**					
WBC, × 10^9^/L	10.17 (6.96, 14.08)	10.78 (7.39, 14.87)	9.94 (7.10, 14.14)	9.68 (6.82, 13.09)	0.257
Neu, × 10^9^/L	8.23 (5.07-12.01)	9.56 (5.84-13.36)	8.47 (5.23-11.50)	7.14 (4.07-10.25)	< 0.001^*^
Lym, × 10^9^/L	0.92 (0.55-1.40)	0.58 (0.39-0.89)	0.90 (0.60-1.18)	1.49 (1.10-2.27)	< 0.001^*^
RBC, × 10^12^/L	4.21 (3.77, 4.69)	3.97 (3.40, 4.51)	4.16 (3.79, 4.55)	4.49 (4.04, 4.92)	< 0.001^*^
Hb, g/L	120.00 (104.00, 134.00)	114.00 (94.00, 128.00)	124.00 (107.50, 133.00)	125.00 (112.00, 139.25)	< 0.001^*^
PLT, × 10^9^/L	205.50 (152.00, 258.00)	175.00 (118.00, 226.00)	200.00 (162.00, 246.00)	236.00 (187.50, 279.25)	< 0.001^*^
CRP, mg/L	72.60 (33.60-111.50)	99.80 (56.40-120.00)	72.50 (34.18-102.65)	51.10 (19.12-77.20)	< 0.001^*^
PT, second	12.60 (11.60, 13.80)	12.90 (12.00, 14.15)	12.80 (11.80, 13.80)	12.00 (11.30, 13.22)	< 0.001^*^
TT, second	13.80 (12.80, 14.80)	13.90 (13.30, 15.10)	13.70 (12.57, 14.50)	13.70 (12.80, 14.90)	0.007^*^
APTT, second	31.95 (29.60, 34.00)	31.40 (28.63, 33.68)	32.10 (30.35, 34.18)	32.10 (29.90, 34.38)	0.099
Fib, g/L	4.37 ± 1.28	4.61 ± 1.32	4.46 ± 1.32	4.01 ± 1.13	< 0.001^*^
TBIL, μmol/L	13.20 (9.20, 19.70)	15.00 (10.30, 23.30)	12.65 (9.00, 18.90)	11.85 (8.90, 18.60)	0.015^*^
DBIL, μmol/L	3.60 (2.20, 7.10)	5.30 (3.00, 10.05)	3.60 (2.20, 6.00)	2.75 (2.00, 5.32)	< 0.001^*^
IBIL, μmol/L	9.30 (6.80, 13.20)	9.30 (6.75, 14.00)	9.25 (6.32, 12.50)	8.90 (6.90, 12.95)	0.764
ALT, U/L	22.00 (14.00, 37.00)	23.00 (14.00, 47.00)	23.00 (15.00, 35.75)	21.00 (13.00, 31.25)	0.163
AST, U/L	28.00 (19.00, 47.00)	33.00 (19.00, 65.50)	30.50 (20.00, 47.75)	22.50 (18.00, 32.00)	< 0.001^*^
TP, g/L	62.70 (57.00, 67.00)	56.40 (51.85, 60.85)	63.40 (59.73, 66.83)	66.70 (63.38, 70.90)	< 0.001^*^
ALB, g/L	35.10 (30.40, 38.50)	29.40 (26.10, 31.40)	35.70 (33.40, 36.98)	40.00 (37.95, 42.02)	< 0.001^*^
GLO, g/L	27.30 (23.70, 31.20)	27.40 (23.90, 31.55)	27.80 (24.38, 31.28)	26.40 (23.08, 30.75)	0.311
Cr, μmol/L	88.00 (67.00, 116.00)	100.00 (72.50, 152.00)	93.00 (69.25, 119.75)	77.00 (62.00, 92.25)	< 0.001^*^
UA, μmol/L	331.00 (250.00, 427.00)	329.00 (242.50, 455.50)	325.50 (257.50, 422.00)	340.15 (260.75, 410.25)	0.979
TC, mmol/L	3.67 (2.87, 4.60)	3.09 (2.54, 3.96)	3.65 (3.01, 4.59)	4.22 (3.40, 5.14)	< 0.001^*^
TG, mmol/L	1.13 (0.84, 1.66)	1.33 (0.94, 1.73)	1.05 (0.77, 1.45)	1.08 (0.81, 1.74)	0.002^*^
HDL-C, mmol/L	0.99 ± 0.40	0.76 ± 0.36	1.01 ± 0.33	1.19 ± 0.38	< 0.001^*^
15.6-7.4,-1.3498pt LDL-C, mmol/L	2.15 (1.56, 2.84)	1.83 (1.37, 2.40)	2.18 (1.56, 2.79)	2.49 (1.87, 3.14)	< 0.001^*^
Immune-inflammatory indices					
PLR	206.96 (132.95–357.35)	293.48 (177.26–464.34)	225.76 (166.67–366.07)	145.91 (108.54–200.21)	< 0.001^*^
15.6-7.4,-1.3498pt NLR	9.34 (4.27–17.99)	17.08 (9.06–25.97)	9.85 (5.26–16.13)	4.04 (2.27–7.41)	< 0.001^*^
Nutritional scores					
COUNT	5.00 (3.00–7.00)	8.00 (7.00–9.00)	4.00 (3.00–5.75)	2.00 (1.00–3.00)	< 0.001^*^
BAR	0.17 (0.12–0.26)	0.27 (0.18–0.41)	0.17 (0.13–0.22)	0.13 (0.10–0.16)	< 0.001^*^
mGPS	1.00 (1.00–2.00)	2.00 (2.00–2.00)	1.00 (1.00–2.00)	1.00 (1.00–1.00)	< 0.001

Median (IQR) for non-normal distribution continuous variables. Percentage (%) for categorical variables. ^*^p < 0.05. PNI, prognostic nutritional index; IQR, interquartile range; CVD, cardio-cerebrovascular diseases; WBC, white blood cell counts; Neu, neutrophil counts; Lym, lymphocyte counts; RBC, red blood cell counts; Hb, hemoglobin; PLT, platelet; CRP, C-reactive protein; PT, prothrombin time; TT, thrombin time; APTT, activated partial thromboplastin time; Fib, fibrinogen; TBIL, total bilirubin; DBIL, direct bilirubin; IBIL, indirect bilirubin; ALT, alanine aminotransferase; AST, aspartate aminotransferase; TP, total protein; ALB, albumin; GLO, globulin; Cr, creatinine; UA, uric acid; TC, total cholesterol; TG, triglycerides; HDL-C, high-density lipoprotein cholesterol; LDL-C, low-density lipoprotein cholesterol; PLR, platelet to lymphocyte ratio; NLR, neutrophil to lymphocyte ratio; COUNT, controlling nutritional status score; BAR, BUN to albumin ratio; mGPS, modified Glasgow Prognostic Score.

### 3.2 Association between PNI and long-term all-cause mortality risk

#### 3.2.1 Restricted cubic spline analysis for nonlinear associations

**Figure 2:**
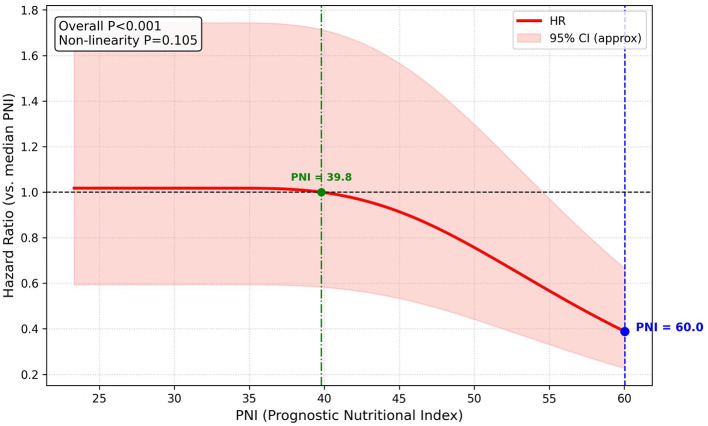
RCS modeling for nonlinear correlation analysis between PNI and long-term all-cause mortality risk. Data were fitted using RCS Cox models with four knots at the 5th, 35th, 65th, and 95th percentiles of PNI, with the 5th percentile as the reference. RCS analysis identified an inverted L-shaped, dose-dependent, nonlinear negative correlation between PNI (inflection point at 39.80) and long-term all-cause mortality risk (*p* < 0.001).

RCS analysis revealed an inverted L-shaped, dose-dependent, nonlinear negative association between lower PNI and higher long-term all-cause mortality risk (*p* for overall < 0.001) (Figure 2). A critical inflection point was identified at PNI 39.8: when PNI fell below this threshold, mortality risk remained consistently elevated and worsened as PNI decreased, indicating that poor nutritional status is directly associated with an increased risk of adverse outcomes. Beyond this inflection point, mortality risk declined rapidly with increasing PNI. Notably, after the inflection point (PNI > 39.8), the shaded 95% confidence interval narrowed considerably, suggesting greater certainty in the mortality risk estimates within this range. These findings underscore the importance of maintaining adequate nutritional levels to improve long-term prognosis in sepsis survivors.

#### 3.2.2 Multivariable cox proportional hazards regression analysis

This study evaluated the association between the PNI and long-term all-cause mortality using multivariable Cox regression analysis (Table 2). The results showed that in the unadjusted model (Model I), both the low-PNI group (hazard ratio [HR] = 3.586, 95% confidence interval [CI] = 2.405–5.347) and the intermediate-PNI group (HR = 1.790, 95% CI = 1.163–2.757) had significantly higher all-cause mortality risks than the high-PNI group (all *p* < 0.01). A significant inverse trend was observed between PNI and all-cause mortality risk (*p* for trend < 0.001). After adjusting for age (Model II), this association remained stable. However, after further adjustment for both age and white blood cell count (Model III), the lowest tertile-PNI group still exhibited a significantly 54.8% higher mortality risk compared with the highest tertile-PNI group (HR = 1.548, 95% CI = 1.016–2.358, *p* = 0.042), whereas the difference between the intermediate- and high-PNI groups became non-significant (*p* = 0.207), and the overall trend was attenuated (*P* = 0.043). In summary, a low PNI is an independent risk factor for long-term all-cause mortality.

**Table 2:** Multivariable cox regression analyses for evaluating the association between PNI and long-term all-cause mortality.

Groups	Overall (Case/total)	HR, 95% CI, and *P*-values					
		Model I	*p*-values	Model II	*p*-values	Model III	*p*-values
High-PNI	33/152	Ref	–	Ref	–	Ref	–
Low-PNI	89/155	3.586 (2.405–5.347)	< 0.001^*^	3.605 (2.417–5.376)	< 0.001^*^	1.548 (1.016–2.358)	0.042^*^
Moderate-PNI	55/154	1.790 (1.163–2.757)	0.008^*^	1.770 (1.149–2.726)	0.010^*^	1.321 (0.857–2.036)	0.207
*P* for trend	–	0.523 (0.431–0.635)	< 0.001^*^	0.520 (0.428–0.632)	< 0.001^*^	0.810 (0.661–0.993)	0.043^*^

Model I: adjusted for none. Model II: adjusted for age. Model III: adjusted for age and WBC count. ^*^p < 0.05. PNI, prognostic nutritional index; HR, hazard ratio; CI, confidence interval; Ref, reference; WBC, white blood cell.

#### 3.2.3 Stratified analysis in subgroup

**Figure 3:**
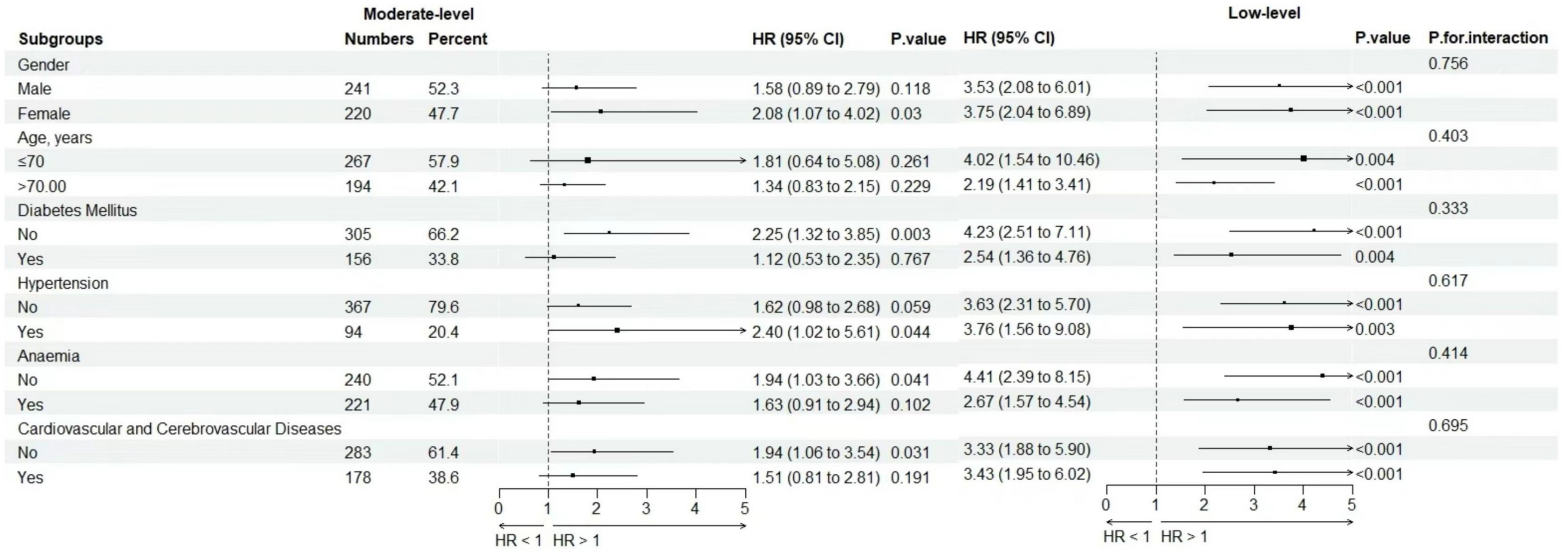
Forest plot for stratified analysis in subgroups. Subgroup-stratified analysis revealed a stable model with consistent predictive value of PNI for long-term all-cause mortality risk across populations, with the high-level group as the reference (all interaction *p*-values > 0.05), supporting its broad clinical applicability.

Subgroup stratification analysis is presented in a forest plot (Figure 3) to assess the association between baseline PNI levels and all-cause mortality, with the high PNI group serving as the reference group (not shown). Results demonstrated that, across most subgroups, the low-PNI group had a significantly higher all-cause mortality risk than the reference group (HR > 1, *p* < 0.05), with hazard ratios ranging from 2.19 to 4.41. In contrast, the moderate-PNI group showed clinically relevant and statistically significant risk increases only in select subgroups, such as females or hypertensive patients. Interaction analyses revealed no significant effect modification by sex, age, diabetes, hypertension, anemia, or cardiovascular disease status (all interaction *p*-values > 0.05), indicating that the relationship between PNI and all-cause mortality remained consistent across these factors. Collectively, these findings support the stable predictive performance and broad clinical applicability of the PNI risk model across diverse patient populations.

### 3.3 Kaplan-Meier survival analysis across the three PNI groups

**Figure 4:**
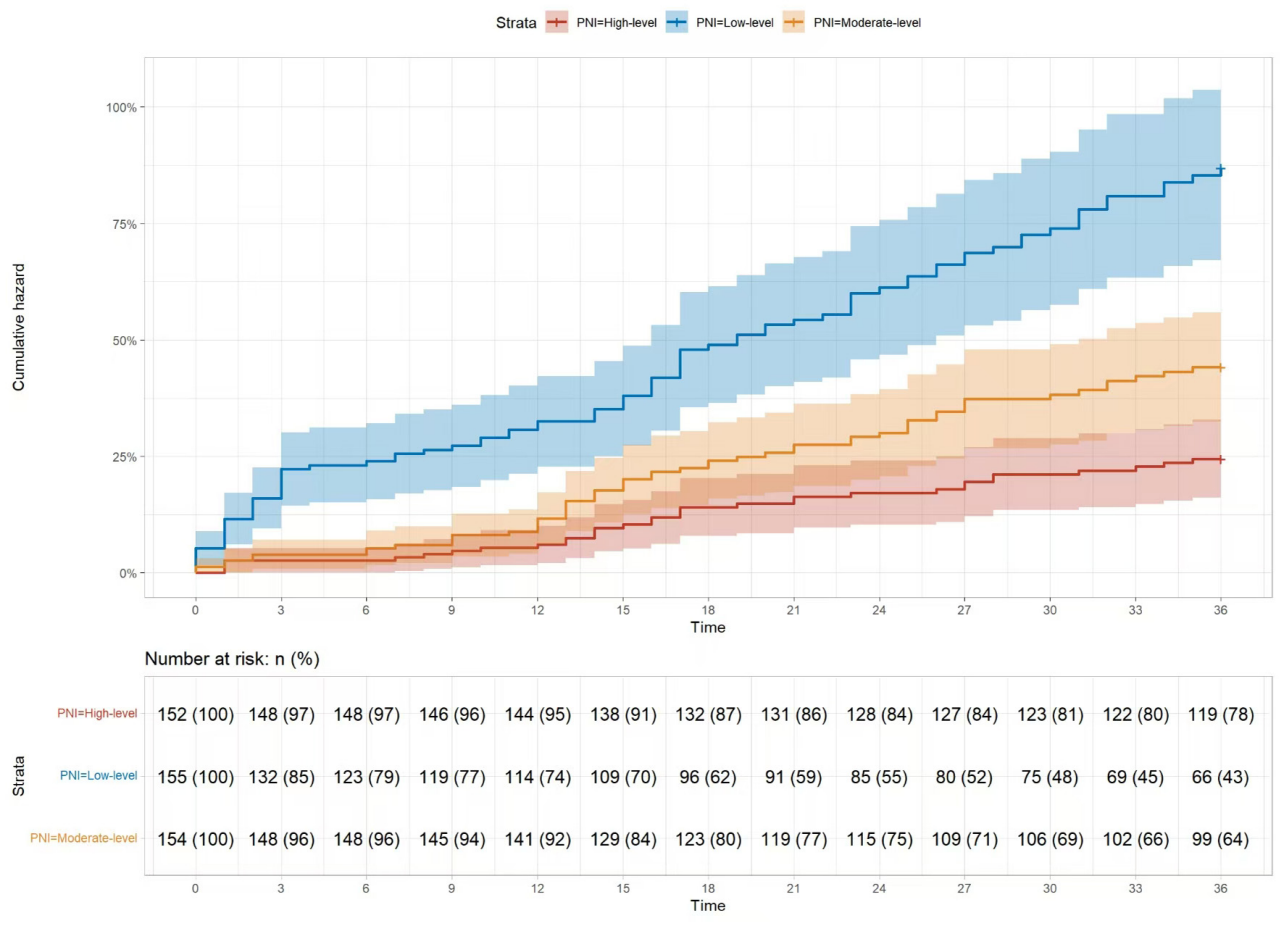
Kaplan–Meier analysis comparing long-term all-cause mortality across the three PNI groups. The survival curves showed a significantly higher cumulative incidence of all-cause mortality in the low-PNI group than in the moderate- and high-PNI groups (log-rank test, *p* < 0.001).

This study conducted a long-term follow-up to evaluate survival outcomes in patients with different PNI levels (Figure 4). Kaplan–Meier curves and number-at-risk data showed that PNI levels were associated with event-free survival. With a median follow-up of 36 months, patients in the high-PNI group had the most favorable survival outcomes, with an estimated 36-month survival rate of 78.29% (119 survivors vs. 33 deaths) and the lowest cumulative all-cause mortality risk over the observation period. In stark contrast, the low-PNI group demonstrated the poorest survival trajectory, characterized by a rapid early decline in survival, reaching only 42.58% at 36 months (66 survivors vs. 89 deceased), along with the highest cumulative all-cause mortality event risk. The moderate-PNI group showed intermediate outcomes, with a 36-month survival rate of 64.29% (99 survivors vs. 55 deceased). Overall, 61.61% (284/461) of enrolled patients were alive at the study's conclusion, corresponding to a total all-cause mortality rate of 38.39% (177/461). Thus, Kaplan–Meier analysis revealed that cumulative survival in the low-PNI group was significantly lower than in both the moderate- and high-PNI groups (log-rank test, *P* < 0.001), suggesting that PNI, as a composite indicator of nutritional and inflammatory status, holds practical prognostic value: higher PNI levels correlate with improved long-term event-free survival and better clinical outcomes, whereas lower PNI levels are associated with a higher cumulative incidence of all-cause mortality events. These findings support the utility of PNI as an indicator of long-term all-cause mortality risk for risk stratification and prognosis assessment.

### 3.4 Comparison of predictive performance between PNI and related nutritional-inflammatory indicators

#### 3.4.1 Multivariate linear regression analysis

A multivariate linear regression analysis examining changes in dependent variables, such as nutritional and immunoinflammatory markers, as a function of the independent variable PNI is shown in Table 3. Multivariate linear regression further revealed that each one-tertile increase in PNI (tertile interval = 5.9) corresponded to a quantifiable reduction (calculated as |β| × 5.9) in related nutritional and inflammatory markers, in model III (adjusted for age, gender, diabetes, and cardio-cerebrovascular diseases). Specifically, these reductions included 1.0585 in COUNT score (β = −0.1794; 95% confidence interval [CI] = −0.1976, −0.1611), 0.1398 in mGPS (β = −0.0237; 95% CI = −0.0291, −0.0183), 0.0378 in BAR (β = −0.0064; 95% CI = −0.0081, −0.0047), 45.6619 in PLR (β = −7.7393; 95% CI = −11.2440, −4.2345), and 2.8951 in NLR (β = −0.4907; 95% CI = −0.6528, −0.3286), respectively (all *p* < 0.001). These results indicate that PNI exhibits a significant negative correlation with the aforementioned nutritional and immune-inflammatory markers, effectively reflecting nutritional and immune-inflammatory status.

**Table 3:** Multivariate linear regression analysis estimating the effect of a one-unit increment in PNI on specific nutritional and inflammatory indices.

Indices (dependent variables)	Model I		Model II		Model III	
	β (95% CI)	*p*-values	β (95% CI)	*p*-values	β (95% CI)	*p*-values
CONUT	−0.1854 (−0.2034, −0.1674)	< 0.001^*^	−0.1807 (−0.1990, −0.1625)	< 0.001^*^	−0.1794 (−0.1976, −0.1611)	< 0.001^*^
BAR	−0.0072 (−0.0089, −0.0054)	< 0.001^*^	−0.0067 (−0.0085, −0.0049)	< 0.001^*^	−0.0064 (−0.0081, −0.0047)	< 0.001^*^
mGPS	−0.0263 (−0.0316, −0.0209)	< 0.001^*^	−0.0239 (−0.0294, −0.0185)	< 0.001^*^	−0.0237 (−0.0291, −0.0183)	< 0.001^*^
PLR	−7.8287 (−11.2276, −4.4299)	< 0.001^*^	−7.7102 (−11.2105, −4.2099)	< 0.001^*^	−7.7393 (−11.2440, −4.2345)	< 0.001^*^
NLR	−0.5270 (−0.6856, −0.3684)	< 0.001^*^	−0.5021 (−0.6647, −0.3395)	< 0.001^*^	−0.4907 (−0.6528, −0.3286)	< 0.001^*^

Model I: adjusted for none. Model II: adjusted for age and gender. Model III: adjusted for age, gender, diabetes, and cardio-cerebrovascular diseases. ^*^p < 0.05. PNI, prognostic nutritional index; CI, confidence interval; COUNT, controlling nutritional status score; BAR, BUN to albumin ratio; mGPS, modified Glasgow Prognostic Score; PLR, platelet to lymphocyte ratio; NLR, neutrophil to lymphocyte ratio.

#### 3.4.2 Receiver operating characteristic curve analysis

**Figure 5:**
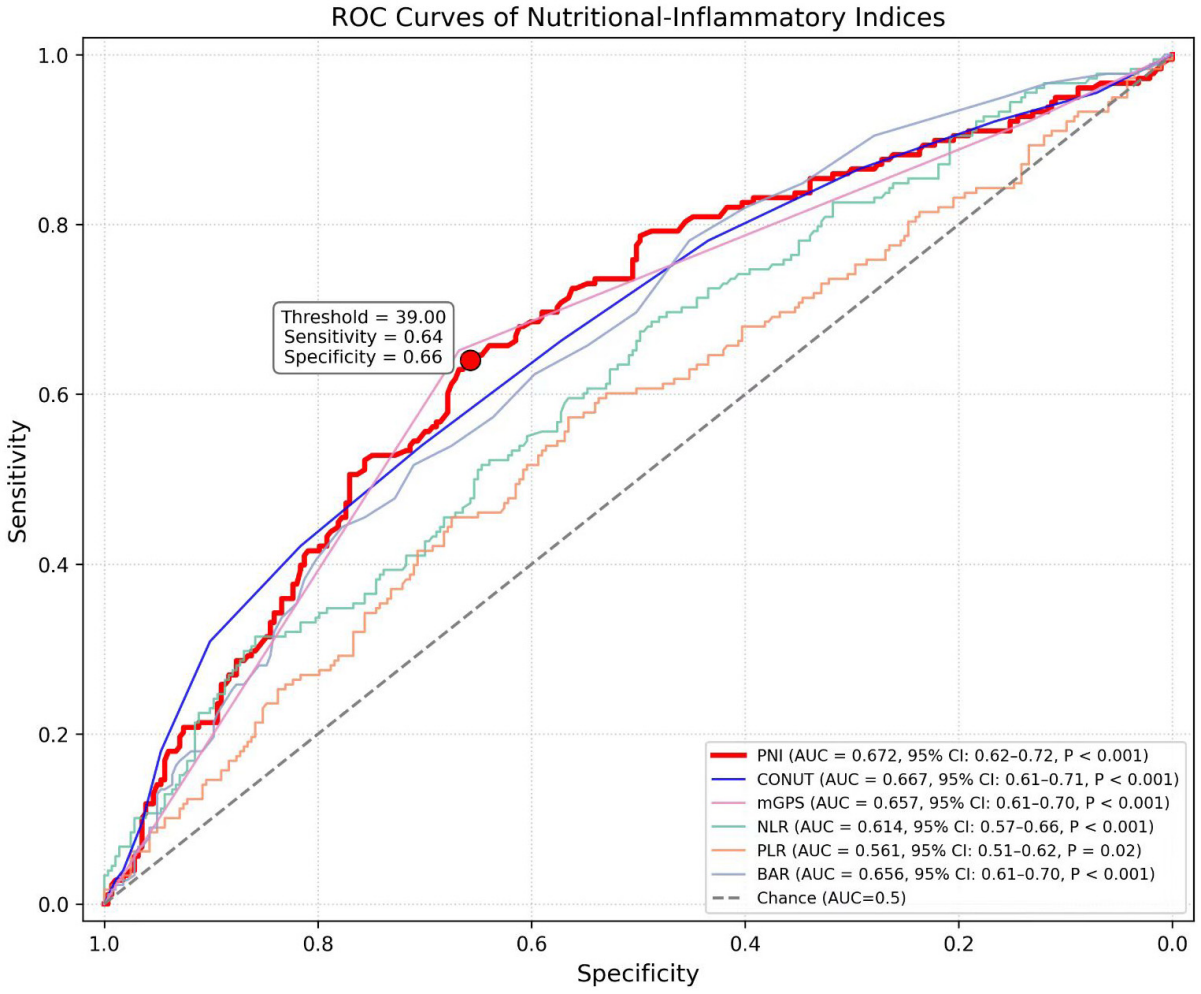
ROC curve analysis for the diagnostic performance of related nutrition-inflammation markers. The ROC curve analysis identified an optimal PNI cutoff of 39.00 for predicting long-term all-cause mortality, with an AUC of 0.672 (95% CI = 0.62–0.72, *p* < 0.001), sensitivity of 0.64, and specificity of 0.66. The prognostic performance of PNI was superior to that of other nutrition-inflammation markers, including CONUT, mGPS, BAR, NLR, and PLR.

The efficacy of PNI and nutritional and immunoinflammatory markers for predicting long-term all-cause mortality is shown in Figure 5. ROC curve analysis identified a PNI clinical diagnostic cutoff of 39.00 for long-term all-cause mortality, with an area under the curve (AUC) of 0.672 (95% CI: 0.62–0.72, *p* < 0.001), sensitivity of 0.64, and specificity of 0.66, which outperformed those of CONUT (AUC = 0.667, 95% CI = 0.61–0.71, *p* < 0.001), mGPS (AUC = 0.657, 95% CI = 0.61–0.70, *p* < 0.001), BAR (AUC = 0.656, 95% CI = 0.61–0.70, *p* < 0.001), NLR (AUC = 0.614, 95% CI = 0.57–0.66, *p* < 0.001), and PLR (AUC = 0.561, 95% CI = 0.51–0.62, *p* = 0.02). The results indicate that nutritional status carries greater weight in long-term all-cause mortality risk than immunoinflammatory markers, suggesting that PNI may serve as an effective prognostic indicator integrating information on both nutritional and immunoinflammatory status.

#### 3.4.3 Internal validation analysis

**Figure 6:**
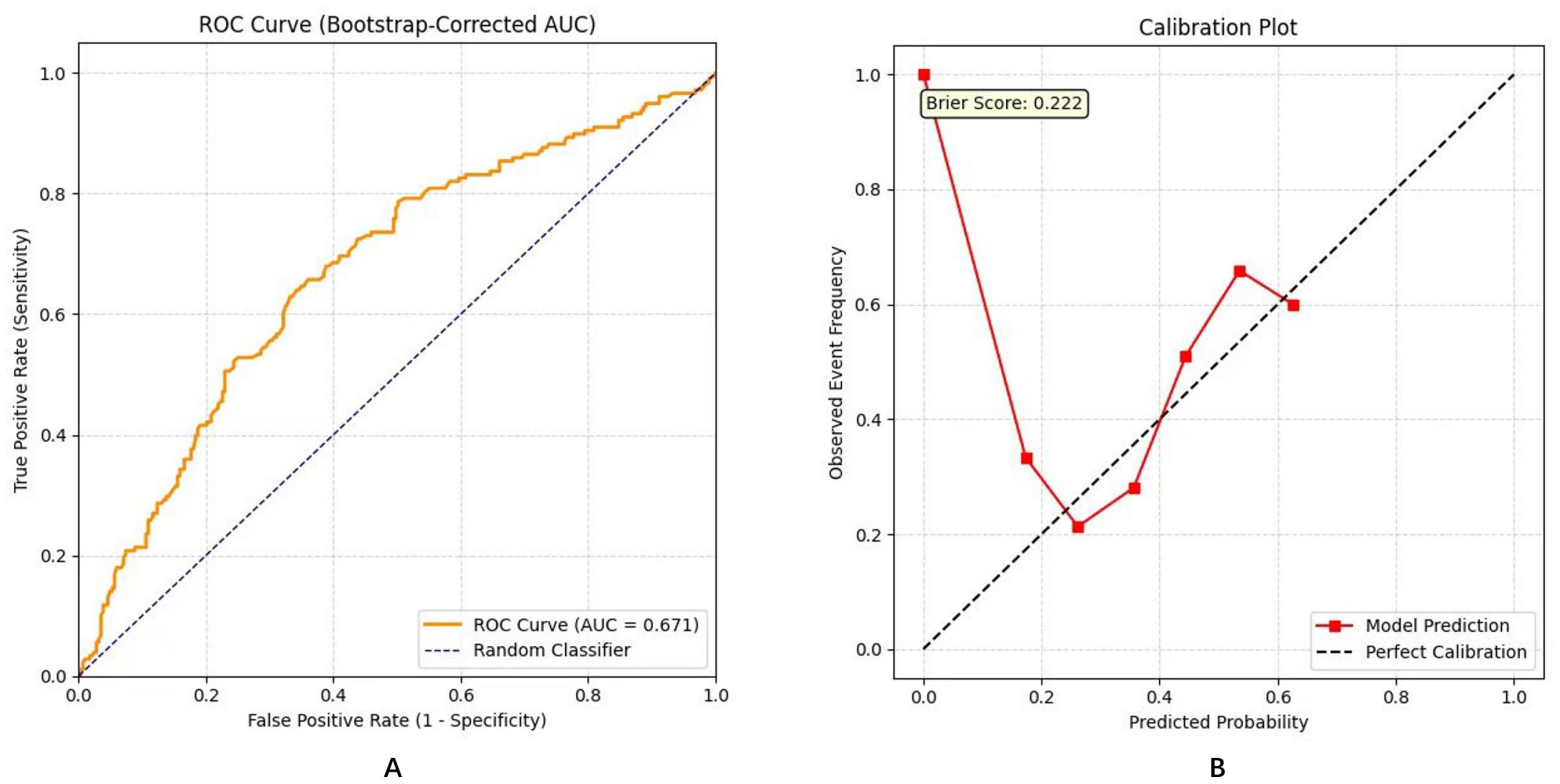
Internal validation of the diagnostic performance of PNI in long-term all-cause mortality. **(A)** The 5-fold cross-validation, combined with a bootstrap-corrected procedure (repeated 1,000 times), was used for internal validation. The 5-fold cross-validation AUC and the bootstrap-corrected AUC were both 0.671 (approximately equal to the original AUC of 0.672), indicating that PNI performs moderately. **(B)** Calibration plot for validating PNI-predicted all-cause mortality risk. Fluctuations in PNI-predicted probability near the 40% threshold reflect slight instability in model calibration in the high-risk range, suggesting that clinical judgment (e.g., related complications) should be considered when interpreting predictions at this risk level. Furthermore, PNI calibration remains well maintained, with a low Brier score of 0.222, supporting PNI as an auxiliary (rather than substitutive) indicator in personalized clinical decision-making and routine care.

Given the low AUC and sensitivity of the PNI on the ROC curve, we conducted internal validation using 5-fold cross-validation with a bootstrap-corrected procedure (1,000 repetitions) and a calibration plot. The 5-fold cross-validation AUC and the bootstrap-corrected AUC were both 0.671 (approximately equal to the original AUC of 0.672) (Figure 6A), indicating that the PNI has a moderate performance. We also validated the performance of the PNI-predicted all-cause mortality risk using a calibration plot (Figure 6B). At the 40% probability threshold commonly used in clinical practice (28), the model's predicted probability showed only mild fluctuations around the ideal calibration line. It exhibited near-perfect calibration within the 20%−60% probability range, indicating that PNI calibration remains well preserved. This is further supported by a lower Brier score of 0.222. These findings reinforce the role of PNI as an auxiliary (rather than a substitutive) indicator for personalized clinical decision-making and routine patient care.

## 4 Discussion

Sepsis survivors often suffer from prolonged adverse clinical outcomes, primarily driven by the PICS, which falls under the category of post-intensive-care syndrome or post-sepsis syndrome (PSS). This is strongly associated with underlying malnutrition and immunoinflammatory conditions (29, 30). It is not merely an excessive inflammatory response but rather a disrupted balance between pro-inflammatory and immunosuppressive arms, with the latter playing a significant role in persistent immune dysfunction and adverse outcomes. The PNI, calculated from serum albumin levels and lymphocyte count, serves as a composite marker reflecting both nutritional and immune status (31, 32). Nevertheless, the relationship between PNI and long-term all-cause mortality risk in sepsis survivors remains inadequately explored (33).

Using a multicenter ambispective longitudinal cohort, this study systematically evaluated the prognostic value of PNI measured at hospital discharge for long-term all-cause mortality among survivors of pneumonia-induced sepsis. In contrast to previous studies that have focused mainly on short-term ICU mortality (34, 35), we extended prognostic assessment into the post-discharge period, with a median follow-up of 36 months. Assessing PNI at discharge helps capture patients' physiological state as they transition from acute care to long-term recovery in home settings. This approach enables the identification of individuals with limited recovery potential despite meeting standard discharge criteria. It provides a rationale for early, individualized interventions, such as tailored nutritional support, immune modulation, and cardiovascular risk management, to improve long-term outcomes.

In this cohort analysis, PNI showed an inverted L-shaped, dose-dependent, nonlinear negative relationship with long-term all-cause mortality: patients in the lowest PNI tertile had a 54.8% higher mortality risk than those in the highest tertile, and risk progressively increased as PNI tertiles declined. PNI was inversely correlated with other nutrition-inflammation markers, including COUNT score, mGPS, BAR, PLR, and NLR, confirming its ability to synthesize prognostic information from both nutritional and inflammatory dimensions. Notably, PNI outperformed other indicators in predicting long-term all-cause mortality. After adjusting for confounders, including age and white blood cell count, multivariate analysis confirmed that low PNI is an independent risk factor for long-term all-cause mortality, a finding consistently supported across subgroups and underscoring its clinical applicability. Kaplan–Meier curves visually reinforced this risk stratification: cumulative survival improved sequentially across low, moderate, and high PNI groups, with mortality reaching 57.42% in the lowest group by the end of follow-up, compared with only 21.71% in the highest group. RCS analysis indicated that all-cause mortality risk rose steadily when PNI fell below approximately 39.8, beyond which risk declined sharply, a threshold closely aligned with the optimal cutoff of 39.00 identified by ROC curve analysis. For clinical guidance, the RCS-determined inflection point (39.8) is preferred because it reflects the true turning point in the continuously changing risk; the ROC-derived cutoff value (39.0) can be used as an adjunct for simple binary screening. When the two values are close, a “warning range, such as 38–40,” can be proposed to guide clinical practice. Moreover, we propose that the PNI at discharge should be viewed as a “snapshot” reflecting current physiological reserve, rather than a permanent imprint of injury. Biologically, both components of PNI are reversible: serum albumin levels are highly sensitive to protein-energy intake and inflammatory burden. They can be improved through nutritional support within a relatively short period. Although lymphocyte counts recover more slowly, existing evidence suggests that immunomodulatory therapies (e.g., thymosin α1, IL-7, etc.) can partially reverse post-sepsis lymphodepletion. Despite the significantly higher mortality risk in the low PNI group at discharge, 43% of patients in this group survived the 36-month follow-up period. This finding strongly suggests that for some patients with low PNI, their physiological function is not in an “irreversible” state. The time window between 3 and 36 months post-discharge may represent a golden period for implementing nutritional and immunological interventions to improve prognosis. Thus, a discharge PNI serves as a simple, quantitative prognostic tool to identify sepsis survivors at substantially elevated long-term post-hospital all-cause mortality risk, enabling timely and proactive clinical intervention.

The reliability of the PNI lies in its combination of two biologically relevant markers, albumin and lymphocyte count, both closely linked to cardiovascular pathophysiology, a key driver of late mortality among sepsis survivors (36, 37). Albumin regulates endothelial function (38), oxidative stress (39), and immunoinflammatory activity (40); hypoalbuminemia reflects not only malnutrition but also systemic inflammation and capillary leakage (41). In this study, the low PNI group exhibited higher direct bilirubin levels, elevated fibrinogen, and prolonged coagulation times, suggesting concurrent liver dysfunction, hypercoagulability, and endothelial injury, all of which are recognized as cardiovascular risk factors. Meanwhile, lymphocytes are implicated in immunosenescence (42), chronic inflammation (43), and plaque stability (44). Persistent lymphocytopenia after sepsis is a hallmark of PICS (45). The low PNI group showed markedly reduced lymphocyte counts together with elevated NLR and PLR, indicating coexisting immunosuppression and sustained inflammation. This milieu promotes atherosclerosis, accelerates cardiovascular aging, and heightens susceptibility to infections and metabolic wasting (46). Thus, a vicious cycle develops over an extended period after sepsis discharge: malnutrition impairs immunity and drives catabolism; immunosuppression increases the risk of infection and inflammation; and chronic inflammation further depletes nutritional reserves and directly damages cardiovascular structures. Essentially, PNI serves as a composite biomarker that captures both nutritional reserves and inflammatory burden.

Building upon prior research demonstrating the prognostic value of the PNI for short-term outcomes in ICU stays in sepsis (typically assessed at admission) (47), this study expands the field by evaluating PNI at discharge, a time point that more accurately reflects the residual physiological state after acute stabilization and before long-term recovery. Focusing on survivors of pneumonia-induced sepsis who remained alive and free of recurrence for over 3 months, we minimized confounding from competing mortality risks. Our analysis demonstrated PNI's superior integrative capacity compared to other common nutrition-inflammation markers and further elucidated its correlations with multiple laboratory parameters. These findings offer mechanistic insights into its association with long-term all-cause mortality risk in sepsis survivors.

In conclusion, PNI at discharge is a practical, integrative biomarker that effectively predicts long-term all-cause mortality in sepsis survivors caused by pneumonia. It reflects the interplay among nutrition, immunity, and inflammation that underlies post-sepsis syndrome and subsequent cardiovascular risk. A discharge PNI range of 38–40 (covering 39.0 by ROC and 39.8 by RCS) can help identify sepsis patients who may benefit from early, proactive long-term management strategies.

### 4.1 Limitations

Several limitations of this study should be acknowledged. First, as an observational investigation, it cannot establish causality between PNI and mortality. Second, although extensive laboratory indicators were analyzed, detailed post-discharge information on medications, rehabilitation, social support, and diet, all potential modifiers of long-term outcomes, was unavailable. Third, PNI was calculated from a single pre-discharge measurement, whereas its dynamic trajectory may provide additional prognostic insight. Fourth, this study employed a retrospective plus prospective follow-up design, and micronutrient levels, such as vitamins, minerals, sodium, potassium, and ferritin reserves, were not systematically collected. Future studies should incorporate a more comprehensive set of nutritional assessment indicators. Finally, the utility of PNI should be further evaluated in sepsis cohorts of varying etiologies and in other critically ill populations.

## 5 Conclusions

These findings indicate that PNI, a marker of the nutrition-immune-inflammation axis, may serve as a cost-effective, practical bedside prognostic tool for assessing the long-term risk of all-cause mortality among survivors of pneumonia-induced sepsis. It may thus support personalized clinical decision-making and routine care as an auxiliary (rather than substitutive) indicator.

## Data Availability

The original contributions presented in the study are included in the article/Supplementary material, further inquiries can be directed to the corresponding authors.
