# Supplementary figures and images for: Prognostic nutritional index at discharge as a practical bedside tool for long-term all-cause mortality risk in pneumonia-induced sepsis survivors
Source: Front Nutr. 2026 May 28;13:1813273. doi: 10.3389/fnut.2026.1813273 (PMC13253304; doi:10.3389/fnut.2026.1813273)

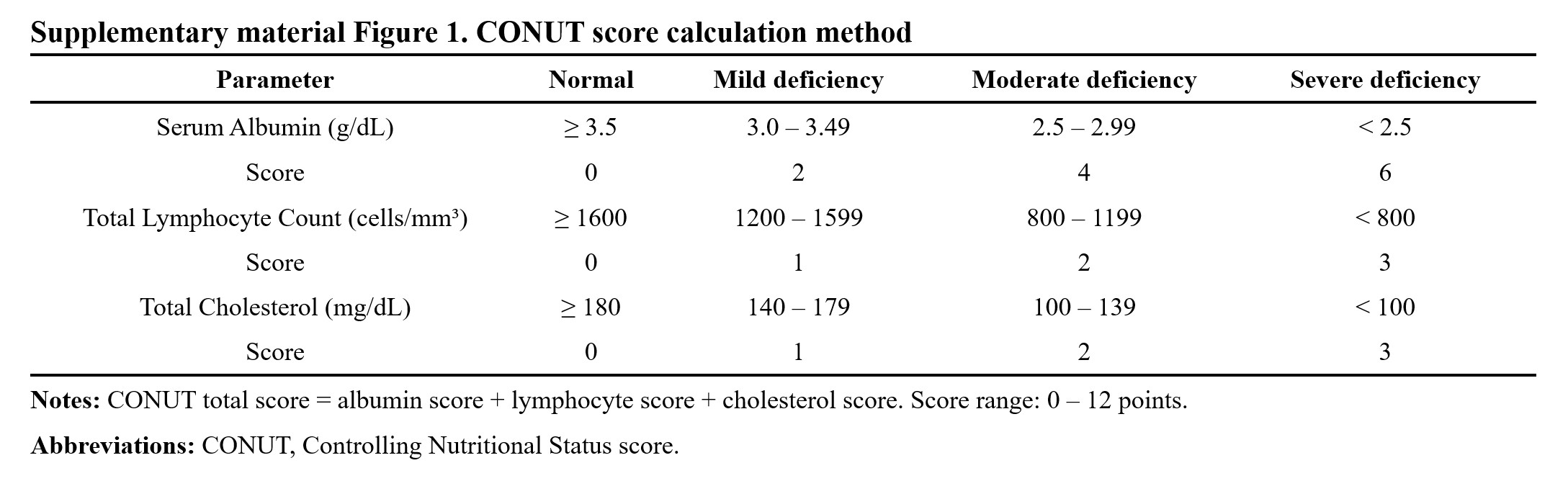

Supplement: Supplementary file 1 [file Image_1.jpeg]
